# Identification of Novel Inhibitor of Enoyl-Acyl Carrier Protein Reductase (InhA) Enzyme in *Mycobacterium tuberculosis* from Plant-Derived Metabolites: An In Silico Study

**DOI:** 10.3390/antibiotics11081038

**Published:** 2022-08-01

**Authors:** Kratika Singh, Niharika Pandey, Firoz Ahmad, Tarun Kumar Upadhyay, Mohammad Hayatul Islam, Nawaf Alshammari, Mohd Saeed, Lamya Ahmed Al-Keridis, Rolee Sharma

**Affiliations:** 1 Department of Biosciences, Integral University, IIRC-3, Kursi Road, Lucknow 226026, Uttar Pradesh, India; kratika.s176@gmail.com (K.S.); niharikapandey19@gmail.com (N.P.); firozz@student.iul.ac.in (F.A.); 2 Department of Biotechnology, Parul Institute of Applied Sciences and Animal Cell Culture and Immunobiochemistry Lab, Centre of Research for Development, Parul University, Vadodara 391760, Gujarat, India; tarun_bioinfo@yahoo.co.in; 3 Department of Biosciences, Integral University, IIRC-5, Kursi Road, Lucknow 226026, Uttar Pradesh, India; hayatbt@iul.ac.in; 4 Department of Biology, College of Sciences, University of Ha’il, P.O. Box 2440, Ha’il 81411, Saudi Arabia; naib.alshammari@uoh.edu.sa (N.A.); mo.saeed@uoh.edu.sa (M.S.); 5 Department of Biology, College of Science, Princess Nourah Bint Abdulrahman University, P.O. Box 84428, Riyadh 11671, Saudi Arabia; 6 Department of Life Sciences and Biotechnology, C.S.J.M.U (Formerly Kanpur University), Kanpur 208024, Uttar Pradesh, India

**Keywords:** FAS-II, gravacridonediol, InhA, molecular docking, Rutaceae family, tuberculosis

## Abstract

*Mycobacterium tuberculosis* (*M.tb*.) enoyl-acyl carrier protein (ACP) reductase (InhA) is validated as a useful target for tuberculosis therapy and is considered an attractive enzyme to drug discovery. This study aimed to identify the novel inhibitor of the InhA enzyme, a potential target of *M.tb*. involved in the type II fatty acid biosynthesis pathway that controls mycobacterial cell envelope synthesis. We compiled 80 active compounds from *Ruta graveolens* and citrus plants belonging to the Rutaceae family for pharmacokinetics and molecular docking analyses. The chemical structures of the 80 phytochemicals and the 3D structure of the target protein were retrieved from the PubChem database and RCSB Protein Data Bank, respectively. The evaluation of druglikeness was performed based on Lipinski’s Rule of Five, while the computed phytochemical properties and molecular descriptors were used to predict the ADMET of the compounds. Amongst these, 11 pharmacokinetically-screened compounds were further examined by performing molecular docking analysis with an InhA target using AutoDock 4.2. The docking results showed that gravacridonediol, a major glycosylated natural alkaloid from *Ruta graveolens*, might possess a promising inhibitory potential against InhA, with a binding energy (B.E.) of −10.80 kcal/mole and inhibition constant (Ki) of 600.24 nM. These contrast those of the known inhibitor triclosan, which has a B.E. of −6.69 kcal/mole and Ki of 12.43 µM. The binding efficiency of gravacridonediol was higher than that of the well-known inhibitor triclosan against the InhA target. The present study shows that the identified natural compound gravacridonediol possesses drug-like properties and also holds promise in inhibiting InhA, a key target enzyme of *M.tb.*

## 1. Introduction

Tuberculosis (TB) is a transmittable respiratory disease caused by the bacteria *Mycobacterium tuberculosis (M.tb)*. The World Health Organization (WHO) has estimated that infectious agent TB was in the top 10 most common causes of death in 2019, with about 10 million people being infected and 1.4 million people dying of TB; approx. 88% of the TB patients were adults and 12% were children [1]. Both TB incidence and TB mortality rates slightly decreased in the Americas and European WHO Region, plus a some countries in the Eastern Mediterranean and Western Pacific regions in 2020, and they did not reach the 2019 milestone [2,3]. When curing TB, the foremost difficulty is controlling the emergence of *M.tb* strains that are resistant to the frontline anti-TB drugs such as isoniazid (INH), rifampicin (RIF), ethionamide (ETA), ethambutol (EMB), and pyrazinamide (PZA) [4]. The failure of these drugs results in multi-drug resistant (MDR) and extensively drug-resistant (XDR) strains [5]. In order to overcome the issues that MDR and XDR strains cause, the discovery and validation of new TB targets in *M.tb.* have become important over the past decades, a route that could help to introduce a new mechanism of action, reduce the standard therapy time, and improve the cost-effectiveness of therapy. One of the key targets of *M.tb*., InhA, is associated with fatty acid biosynthesis, which is essential for organisms [6]. The enoyl reductase InhA belongs to a family of short-chain reductase, which catalyzes the NADH-specific reduction of 2-trans-enoyl-ACP in the final elongation step of the fatty acid synthases-II (FAS-II) system, occurring in the mycolic acid pathway using NADH as a co-factor [7]. It was clinically proven that INH, ETA, and triclosan inhibit InhA [8].

Medicinal plants serve as a substitute for conventional drugs in the treatment of tuberculosis. These plants are natural resources that have been essential to cultures and play a key role in human lifestyle [9,10]. Recently, it was reported that the Asiatic acid from *Centella asiatica* is a promising compound with the highest inhibitory potential against the *M.tb* GlgE target, which is involved in the FAS-II pathway [11]. Before the invention of modern medicine, the plant was used to treat many diseases, and this practice has continued into today, with the plant being used as the basis for the formation of new drugs and pharmaceutical products [12]. The Rutaceae family of plants has been known for their economic value, representing a potential source of natural active principles. Ruta is the most investigated genera of this family [13]. The *Ruta graveolens* plant was used in the early days, and herbal medicine belongs to the Rutaceae family [14]. Not only have cough, diphtheritic laryngitis, colic, and headache been relieved by rue, but it has also been used as an antidote for poison spread by mushrooms, snake bites, and insect bites [15]. The citrus genus of the Rutaceae family contains 130 genera in the seven subfamilies, comprising many important fruits and essential oil producers [16]. The citrus plant has been widely used and is still commonly used in folk medicine. *Citrus limon L. (C. limon),* Burmese fruit, is employed as tooth powder to take care of orodental health [17]. It was reported that *Citrus lemon* and *Ruta graveolens* of the Rutaceae family were used as traditional medicine for the treatment of *M.tb.* in South Africa [18] and Tamil Nadu (India) [19].

In the present study, InhA inhibitors from the bioactive compounds of Rue (*Ruta graveolens*) and citrus, two common plants that belong to the Rutaceae family, were evaluated through in silico study. The drug likeliness and pharmacokinetics of the bioactive compound were also evaluated with respect to their drug-like nature.

## 2. Material and Methods

### 2.1. Compilation of Bioactive Compounds from Ruta Graveolens and Citrus of Rutaceae Family

We compiled 80 bioactive compounds from the Rutaceae family, of which 44 were from citrus plant and 36 from *Ruta graveolens*. These were retrieved from the literature and Dr. Duke’s Phytochemical and Ethno botanical Databases [20].

### 2.2. Drugs Target Identification and Ligand Preparation

The InhA target protein of *M.tb.* was downloaded from the Protein Data Bank (PDB ID: 1BVR) [21] and saved in pdb format. Hetero-atoms, water molecules, and ligands attached to the downloaded pdb file were removed by using BIOVIA Discovery Studio Visualizer 2020 (BIOVIA, Dassault Systèmes; https://discover.3ds.com/discovery-studio-visualizer-download, accessed on 12 October 2021). The 3D-SDF file format of ligands was downloaded from PubChem database (http://pubchem.ncbi.nlm.nih.gov, accessed on 12 October 2021) and further converted in PDB file format via BIOVIA Discovery Studio Visualizer.

### 2.3. Drug-Likeliness Study of the Compounds

The selected phytochemicals were checked for their drug-likeliness properties against the Lipinski Rule of Five, formulated by Christopher A. Lipinski in 1997 [22]. Lipinski’s filters were applied using Molinspiration (http://www.molinspiration.com/, accessed on 12 October 2021) for examining the drug-likeness attributes of the compounds, including the quantity of hydrogen acceptors (<10), hydrogen donors (<5), molecular weight (<500 Daltons), and partition coefficient log P (<140).

### 2.4. ADME/Tox Profiling of the Filtered Compounds 

Statistically, ADME is the main reason for the failure of many drug candidates during the clinical test. Due to this, ADME properties are considered important parameters for researchers when selecting compounds as drug candidates. The pharmacokinetics (ADME/T) of each compound, in terms of the adsorption, distribution, metabolism, excretion, and toxicity, were evaluated by using an online PreADMET server (http://preadmet.bmdrc.org/, accessed on 12 October 2021). Compounds that followed all the ADMET parameters were selected for the molecular docking analysis. 

### 2.5. Molecular Docking

The ligand–target interaction was evaluated in terms of the binding affinity of the selected compounds against *M.tb.* InhA was evaluated based on molecular docking analysis by using AutoDock 4.2 software (Auto Dock 4.2, Scripps Research, La Jolla, CA, USA, https://autodock.scripps.edu/downloaded, accessed on 12 October 2021). The interaction of the ligands and targets was based on the Lamarckian genetic algorithm (for optimal conformation, a maximum of 10 conformers were considered for each compound) for estimating the binding energy and inhibitory constant [23,24,25]. For InhA targets, the grid was prepared as 70 × 70 × 70 by adjusting the grid on the X, Y, and Z coordinates to 19.050, 18.992, and 9.686, respectively, in the active site of InhA, as we positioned the grid on the active site reported for PDB, while other parameters were set as default for the molecular docking study. The docked complex in the lowest energy cluster was appraised for all later interaction studies. The binding energy and inhibition constant (Ki) are expressed as kilocalories per mole and micro molar (µM). The lead compound was compared with the known inhibitor in terms of its binding with the target protein. The Tanimoto coefficient was used to analyze the similarity of both the compounds, i.e., the known inhibitor triclosan and gravacridonediol. The Tanimoto coefficient is defined as c/(a + b + c), which is the proportion of the features shared among two compounds divided by their union.

### 2.6. Molecular Dynamics Simulation

The academic Maestro-Desmond v5.6 suite was used for the molecular dynamic simulation of the selected complex from the CC place the docked complex, and the system builder module was used to form an open TIP4P water model. Secondly, the counter sodium and chloride ions used to neutralize the complete system were placed in the binding pocket of the docked ligand, faced at a distance of 20 Å, and the minimization tool was used to minimize under the default parameters. Next, the Martyna–Tobias–Klein barostat and Nose–Hoover thermostat methods were used to keep an NPT ensemble [26]. Under the default parameters, the temperature was set to 1.013 bar and 300 K to simulate the whole system. To calculate the long-range electrostatic interactions in the simulations [27], the particle mesh Ewald method was used, and a cut-off at 9.0 Å was set in the Coulomb interaction. Under optimized potentials for liquid simulations (OPLS), each complex was simulated for 100 ns, and a total of 5000 frames and 2005 force-field parameters were saved for analysis. For the statistical parameters, the generated trajectories of InhA were analyzed along with gravacridonediol and triclosan, along with analyzing the root mean square fluctuation (RMSF) and root mean square deviation (RMSD). Intermolecular interactions (protein–ligand contact mapping) were also analyzed as a 100 ns utility by the Simulation Interaction Diagram (SID) tool, which was applied in the free academic Desmond module using the Maestro-Schrödinger Suite 2018–4 interface [28,29].

## 3. Results

### 3.1. Compilation of Bioactive Compounds

We compiled 80 compounds of different classes from the Rutaceae family. Among the compounds, most of them belonged to the flavonoid class, while the rest were from the alkaloid, coumarin, alcohol, phenol, ketone, hydrocarbon, fluoroquinolone, lipid, carotenoid, and benzoid classes (Supplementary Table S1).

### 3.2. Drug-Likeliness Study of Compiled Compounds

The drug-likeness property of a chemical compound was determined by Lipinski’s Rule of Five (also known as a rule of thumb) to check whether a compound possessing certain pharmacological activity is orally active in humans. The rule states that the molecular mass amount should be ≤500, Clog P ≤ 5, H-bond donors (HBD) ≤ 5, H-bond acceptors (HBA) ≤ 10, and rotational bonds ≤ 10. If there is only one violation (or none at all), it means that the compound can easily bind to the target receptor [30]. If more than two parameters are not within range, a candidate drug is often discarded from further screening [31]. Out of the 80 screened compounds of the Rutaceae family, only 59 compounds were filtered through Lipinski’s Rule of Five (Supplementary Table S2) and were taken further for ADMET profiling. The list of the filtered compounds is given in Table 1.

### 3.3. ADMET Study of Screened Compounds Compared with Standard Drug 

The results of the ADMET profiling showed that only 11 compounds fulfilled all of the ADMET descriptors (Supplementary Table S3). The 11 compounds were in agreement with the drug ability and pharmacokinetic parameters, and were selected for molecular docking analysis (Table 2).

### 3.4. Molecular Docking 

**Figure 1:**
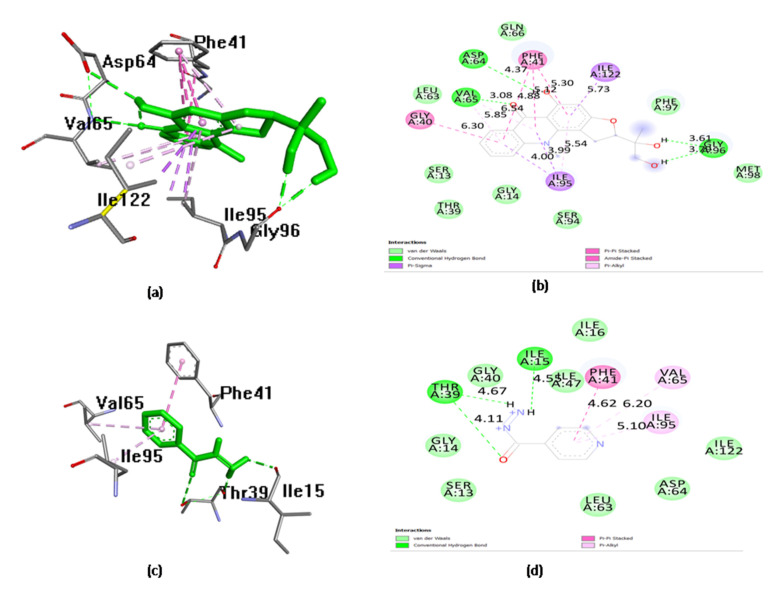
(**a**) Docked pose of gravacridonediol in the InhA binding site, showing the molecular interactions—hydrogen and hydrophobic bonds noted using green and pink/purple dashed lines, respectively; (**b**) 2D plot of the interactions between gravacridonediol and key residues of InhA, generated by BIOVIA Discovery Studio visualizer; (**c**) docked pose of triclosan in the InhA binding site, showing molecular interactions—hydrogen and hydrophobic bonds noted using green and pink/purple dashed lines, respectively; (**d**) 2D plot of interactions between triclosan and key residues of InhA, generated by BIOVIA Discovery Studio visualizer.

Molecular docking analysis conducted with Lipinski and ADMET filters simulated the interactions of the 11 compounds against *M.tb.* target InhA. In this study, triclosan (a known inhibitor of InhA) was used as the reference inhibitor compound of InhA [32,33,34]. Almost all of the compounds showed binding affinity toward the *M.tb.* target InhA (Table 3). The molecular docking results showed that, among all of the tested compounds, gravacridonediol exhibited the best binding affinity with the InhA target. Gravacridonediol showed the lowest binding energy (−10.80 kcal/mole) and inhibition constant (Ki) (600.24 nM) compared with the known inhibitor triclosan, which showed a binding energy of −7.33 kcal/mole and Ki 4.26 µM (Table 3). Gravacridonediol exhibited a 1.5-fold higher binding affinity compared with the known inhibitor triclosan. The binding mode and interactive amino acids of the InhA active site with gravacridonediol are shown in Figure 1. Furthermore, the pharmacokinetic results of gravacridonediol were also satisfactory; in terms of its toxicity, it is non-mutagenic and non-carcinogenic. Gravacridonediol showed a 100% HIA value, which is in agreement with better intestinal absorption.

### 3.5. Analysis for Molecular Dynamics Simulation 

**Figure 2:**
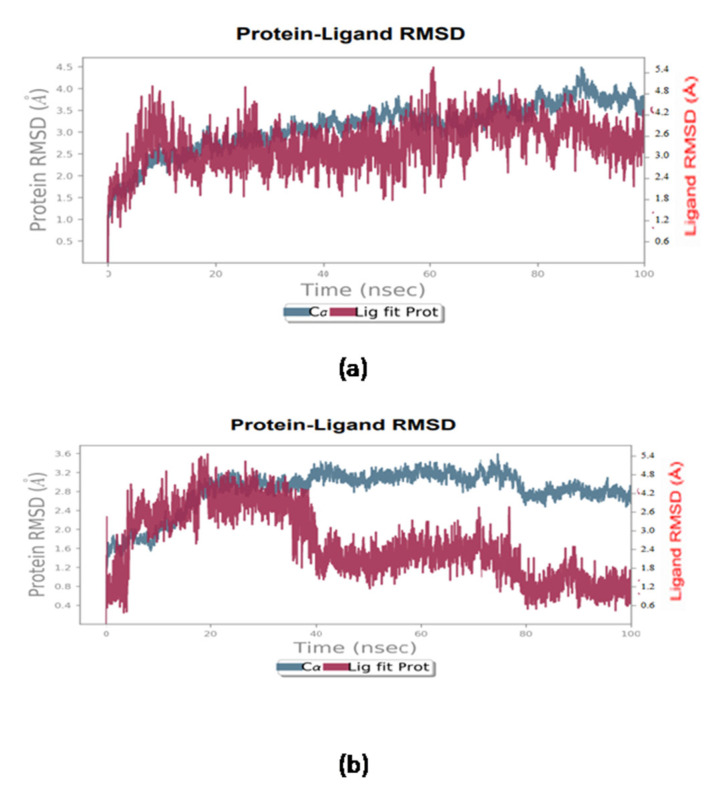
RMSD plot for the docked complex of (**a**) Gravacridonediol-InhA (**b**) Triclosan- InhA.

With the help of molecular dynamics, the prediction of the protein–ligand complexes stability was attained in the simulation interval. From the molecular dynamic simulations, protein and protein-fit-ligand root mean square deviation (RMSD), protein and protein-fit-ligand root mean square fluctuation (RMSF), as well as protein–ligand contact mapping as a function of 100 ns interval, were analyzed in this study. Furthermore, acceptable deviations (<4Å) with respect to the MD simulation of 100 ns were indicated by comparing it to known compounds and by calculating the protein’s RMSD in all docked complexes with natural compounds. The calculated RMSF values (Supplementary Figure S1), except for occasional higher residual fluctuations (<4Å) in the protein structures docked with their respective compounds, were also observed in the terminal regions. Residues in direct communication with the docked ligands or residues adjoined to the interacting residues also supported these observations. Moreover, considerable deviations (<4.5Å) during the 100 ns simulation interval, in addition to the known compound triclosan (<4.0Å) (Figure 2), were shown by the calculated protein-fit-ligand RMSD values. Against known compounds such as triclosan, the most considerable stability and equilibrium in protein-fit-ligand values as a function of the 100 ns interval were also shown by gravacridonediol, among the selected natural compounds. Moreover, the variation within 2 Å (Supplementary Figure S2) during the 100 ns MD simulation and the observed docked ligands stability were also shown by the calculated RMSF values.

Moreover, from all of the docked complexes’ 100 ns MD simulation trajectories, the protein–ligand contact was also extracted. During the 100 ns interval, a percentage of the total molecular contact was created for all simulated complexes, and each type of interaction, including hydrogen bonding, intermolecular, hydrophobic, ionic, and formation of a water bridge, was extracted as well as plotted.

**Figure 3:**
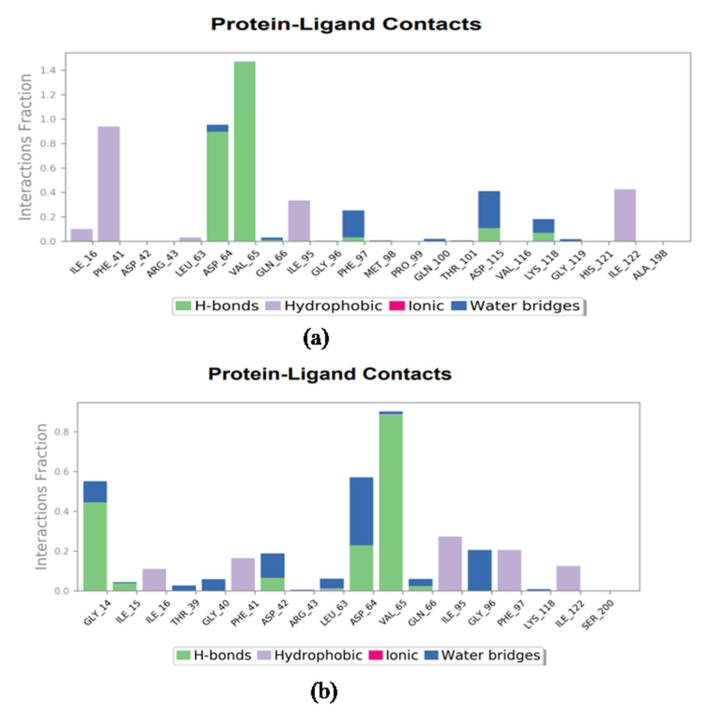
Protein–ligand interaction plots for (**a**) gravacridonediol and (**b**) triclosan.

The gravacridonediol–InhA docked complex formed hydrogen bonds for more than for more than 100% of the simulation time in theVal65 residue and more than 50% of the time in the Leu63 residue. At the 100% simulation interval, a hydrophobic interaction was shown with the docked ligand Phe41. Water bridges were involved in the formation of more than 50% of the hydrogen bonds (Figure 3) in the Phe97 and Asp115 residues. In comparison, the formation of hydrogen bonds accounted for more than 100 percent of the total interaction fraction during the simulation interval, and was shown by the triclosan–InhA docked complex and Val65 and Gly14 residue; Gly14 and Asp64 also participated in water bridge formation (50%) during the simulation interval. A hydrophobic interaction was noted with the docked ligand Ile95 residue for more than 50% of the simulation time (Figure 3, protein–ligand contact graph).

To monitor the stability, flexibility, and folding of the protein in the presence and absence of ligands (Supplementary Figure S3) [35], the solvent accessible surface area (SASA) of the reference molecules and natural compound was also investigated.

Collectively, the selected natural compounds were confirmed to be inhibitors of InhA by the analysis of the MD simulations, compared with the known compound triclosan. We can say that gravacridonediol exhibits the most stability with the InhA target, based on the intermolecular interaction profiling and statistical analysis of the natural compounds that were docked.

## 4. Discussion and Conclusions

*M.tb.* InhA is the fourth enzyme of the FAS-II pathway and is involved in fatty acid biosynthesis [36]. The inactivation of the enzyme causes the agglomeration of fatty acid, the disintegration, and alteration of the cell wall, ultimately leading to pathogen death. Previously, it was reported that InhA is essential for mycolic acid synthesis in *Mycobacterium* [37]. This enzyme was considered a target for the discovery of a novel antitubercular compound due to its biological importance in the FAS-II pathway and structural difference in *M.tb.* and human homologue [38,39]. INH, a commercially available first-line antituberculosis drug, leads to the death of *M.tb.* by inhibiting the function of InhA [40,41,42]. Catalase-peroxidase was involved in the activation of isoniazid in wild-type strains, but due to the mutation in KatG (the gene of catalase-peroxidase), *M.tb.* became MDR and XDR to both isolates [43]. The present study was designed to discover a potent novel inhibitor of InhA.

The results of the present study revealed the compound gravacridonediol as a potent inhibitor of the InhA target, based on binding affinity. Gravacridonediol is an alkaloid isolated from the plant *Ruta graveolens*, which is known as common rue, a widely used medicinal plant. Previously, gravacridonediol has been reported for anti leishmanial, antifungal [44,45], anti-proliferative, and anticancer effects [46,47].

Gravacridonediol showed binding affinity toward the InhA target by H-bonding, π–π interaction, and other bindings. Amino acids of the InhA target Ile194, Pro193, Val 65, and Gly14 were found to interact with gravacridonediol (Table 3). Furthermore, the stability of the complex checked by the MD simulation Val65 was also found to interact with the known inhibitor triclosan by means of H-bonding (Figure 3). Other amino acid residues of the active site, such as Ile21, Ile202, Gly192, Met199, Leu207, Phe149, Tyr158, Ala157, Gly104, and Met103, were also found to interact with the compound. Most of the similar amino acid residues of gravacridonediol and triclosan were found to interact with the target, but gravacridonediol showed a higher binding affinity than the known inhibitor triclosan, with the two having binding energies of −10.49 and −5.49 Kcal/mol, respectively.

**Figure 4:**
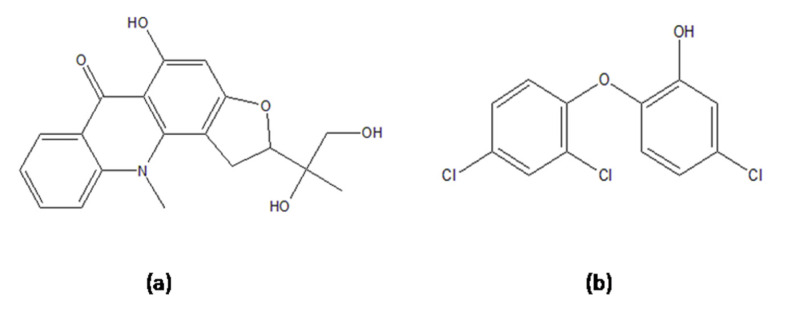
Chemical structure of (**a**) gravacridonediol and (**b**) triclosan (COc1ccccc1).

The similarity of both compounds was calculated by the online ChemMine tool (https://chemminetools.ucr.edu/similarity/, accessed on 17 January 2022), using the Canonical SMILES of both compounds. The Tanimoto coefficient was recorded as 0.16577 after the comparison of both molecules. The MCS Tanimoto coefficient was recorded as 8, which indicates the slight similarity of both molecules. The chemical structure of both molecules (Figure 4) may help the lead compounds bind with the target protein.

**AP Tanimoto**: 0.165775

**MCS Tanimoto**: 0.2353

**MCS Size**: 8

**MCS Min**: 0.4706

**MCS Max**: 0.3200

**SMILES**: COc1ccccc1

Gravacridonediol=C1=CC(=C(C=C1Cl)O)OC2=C(C=C(C=C2)Cl)ClC1=CC(=C(C=C1Cl)O)OC2=C(C=C(C=C2)Cl)Cl

Triclosan= CC(CO)(C1CC2=C(O1)C=C(C3=C2N(C4=CC=CC=C4C3=O)C)O)O

Drug resistance in *M.tb.* has become a major health problem. It has been estimated that 7.2% of TB cases are INH-resistant worldwide and 11.6% in previously treated TB cases [48]. In addition, INH resistance levels can reach up to 60% in previously treated TB cases. The key target of INH is InhA; yet, the main mechanism of INH resistance in TB clinical isolates is the process of mutations in the activator of INH, KatG. *M.tb.* enoyl-reductase InhA participates in mycolic acid biosynthesis. Thus, the final observation after MD simulation revealed that gravacridonediol could be a novel and potent inhibitor of the InhA target of *M.tb.* to inhibit mycolic acid and long-chain fatty acid biosyntheses in *Mycobacterium* as it confirms the stability of the protein–ligand complex. Furthermore, validation of in silico data through in vitro investigations is in the pipeline, to explore the details about the mechanism of cell wall mycolic and long-chain fatty acid biosynthesis inhibition in *Mycobacterium*.

## Figures and Tables

**Table 1:** Screened compounds following the drug-likeliness test.

S. No.	Name of Compound	PubChem ID	Molecular Weight (g/mol) (≤500)	Xlog P3 (≤5)	H-Bond Donor (≤5)	H-Bond Acceptor (≤10)	Rotational Bond (≤10)	n VIOLATION
1.	α-Limonene diepoxide	232703	68.23	1.1	0	2	1	0
2.	Nobiletin	72344	402.4	3	0	8	7	1
3.	Sinensetin	145659	372.4	3	0	7	6	1
4.	Tangeretin	68077	372.4	3	0	7	6	1
5.	Diosmetin	5281612	300.26	1.7	3	6	2	0
6.	Graveoline	353825	279.29	3.1	0	4	1	0
7.	Rutacridone	5281849	307.3	4.6	1	4	1	0
8.	Daphnoretin-methyl-ether	5318544	366.3	3.6	0	7	4	0
9.	Rutaretin	44146779	262.26	1.6	2	5	1	0
10.	Gravacridonediol	5317836	341.4	2.3	3	6	2	0

**Table 2:** ADMET profiling of the filtered compounds.

S.No.	Compound Name	Toxicity		Absorption				Distributions		Metabolism Cyp 2d6
		Mutagenicity (Ames Test)	Carcinogenicity	HIA%	Pcaco-2 (nm/s)	Pmdck (nm/s)	Pskin (nm/s)	PPB%	BBB%	
1.	α-Limonene diepoxide	Mutagenic	Non-carcinogenic	100	57.6	28.79	−2.5	54.15	0.23	Non-inhibitor
2.	Nobiletin	Mutagenic	Non-carcinogenic	99.07	54.02	0.06	−3.6	84.85	0.02	Non-inhibitor
3.	Sinensetin	Mutagenic	Non-carcinogenic	98.8	51.22	0.06	−3.5	86.24	0.02	Non-inhibitor
4.	Tangeretin	Mutagenic	Non-carcinogenic	98.8	53.6	0.62	−3.4	87.17	0.02	Non-inhibitor
5.	Diosmetin	Mutagenic	Non-carcinogenic	88.18	7.02	23.85	−4.1	90.16	0.2	Non-inhibitor
6.	Graveolinine	Mutagenic	Non-carcinogenic	97.83	56.13	37.77	−3.47	89.96	0.04	Non-inhibitor
7.	Guaiacol	Mutagenic	Non-carcinogenic	96.47	29.44	362.86	−1.9	99.18	0.9	Non-inhibitor
8.	Rutacridone	Mutagenic	Non-carcinogenic	95.74	35.63	8.75	−3.28	89.6	0.87	Non-inhibitor
9.	Daphnoretine-methyl-ether	Mutagenic	Non-carcinogenic	99.11	25.71	0.42	−3.65	87.02	0.11	Non-inhibitor
10.	Rutaretin	Non-Mutagenic	Non-carcinogenic	90.96	5.84	253.44	−3.18	72.17	0.59	Non-inhibitor
11.	Gravacridonediol	Non- Mutagenic	Non-carcinogenic	100	19.17	45.96	−4.02	79.22	0.26	Non-inhibitor

HIA, human intestinal absorption; PPB, plasma protein binding; BBB, blood–brain barrier; p, permeability.

**Table 3:** Binding energy of docked protein InhA and ligands, along with that of the natural inhibitor triclosan.

S.No.	Plants	Compound	Binding Energy	Ki (Inhibition Constant)	H-Bond Interacting Amino Acids within a Distance of 3Å	Distance (Å)
1.	Citrus	α-Limonene diepoxide	−4.95	235.01µm	Ile194	2.8574
2.	Citrus	Nobiletin	−6.87	9.19 µm	Ile194	3.13498
3.	Citrus	Sinensetin	−7.98	1.42 µm	________	
4.	Citrus	Tangeretin	−7.04	6.96 µm	Ile 194, Tyr 158	2.74349 2.53957
5.	Citrus	Diosmetin	−7.92	1.98 µm	Ile194, Pro156	2.01273 1.89617
6.	*Ruta graveolens*	Daphnoretin-methyl-ether	−7.62	2.61µm	Ile21, Ala22	2.64217 2.99582
7.	*Ruta graveolens*	Gravacridonediol	−10.49	600.24 nm	Val 65, Gly 96	2.92375 1.98401
8.	*Ruta graveolens*	Graveolinine	−7.69	2.33 µm	Ile 194	2.7123
9.	*Ruta graveolens*	Gvaiacol	−4.09	996.61 µm	Tyr158, Pro 156	2.99138 1.9058
10.	*Ruta graveolens*	Rutacridone	−7.43	3.59 µm	______	
11.	*Ruta graveolens*	Rutaretin	−7.23	5.01 µm	Pro156	2.33402
12.	Drugs	Isoniazid	−5.49	549.74 µm	Thr39, Ile15, Gly14	2.9347 2.09599 2.9347
13.	Natural inhibitor	Triclosan	−6.69	12.43 µm	Gly 14, Thr 39, Ile 15	2.89647 1.92469 2.22936

## Supplementary Materials

The following are available online at https://www.mdpi.com/article/10.3390/antibiotics11081038/s1, Figure S1: RMSF plot for the InhA docked with (a) Gravacridonediol (b) Triclosan, Figure S2: RMSF plot for (a) Gravacridonediol (b) Triclosan, Figure S3: Solvent accessible surface area (SASA) of (a) Gravacridonediol (b) Triclosan, Table S1: Compilation of compounds, Table S2: Screening of compounds for drug-likeliness property, Table S3: ADMET profiling of all the screened compounds.

## Abbreviations

*M.tb*., *Mycobacterium tuberculosis*; ACP, enoyl-acyl carrier protein; InhA, enoyl-acyl carrier protein reductase; WHO, World Health Organization; MDR, multi drug resistance; XDR, extensive drug resistance; HIA, human intestinal absorption; PCaCO2, cell permeability Caco-2 in vitro; PMDCK, cell permeability of Madin–Darby canine kidney; P_Skin_, skin permeability; PPB, plasma protein binding; BBB, blood–brain barrier; MW, molecular weight; FAS-II, fatty acid synthases-II; INH, isoniazid; RIF, rifampicin; ETA, ethionamide; EMB, ethambutol; and PZA, pyrazinamide.
