# Supplementary material for: Identification of Novel Inhibitor of Enoyl-Acyl Carrier Protein Reductase (InhA) Enzyme in Mycobacterium tuberculosis from Plant-Derived Metabolites: An In Silico Study
Source: Antibiotics (Basel). 2022 Aug 1;11(8):1038. doi: 10.3390/antibiotics11081038 (PMC9405319; doi:10.3390/antibiotics11081038)
Supplement: Supplementary file 1 [file antibiotics-11-01038-s001.zip › antibiotics-1810591-supplementary.pdf]

Identification of Novel Inhibitor of Enoyl-Acyl Carrier Protein Reductase (InhA) Enzyme in *Mycobacterium tuberculosis* From Plant-Derived Metabolites: An *In Silico* Study

Kratika Singh [1], Niharika Pandey [1], Firoz Ahmad [1],Tarun Kumar Upadhyay [2], Mohammad Hayatul Islam [3],
Nawaf Alshammari [4], Mohd Saeed [4], Lamya Ahmed Al-Keridis[5,*] and Rolee Sharma [1,6,*]

[1] Department of Biosciences, Integral University, IIRC-3, Kursi Road, Lucknow 226026, Uttar Pradesh, India;
[2] Department of Biotechnology, Parul Institute of Applied Sciences and Animal Cell Culture and Immunobiochemistry Lab, Centre of Research for Development, Parul University, Vadodara 391760, Gujarat, India; tarun_bioinfo@yahoo.co.in
[3] Department of Biosciences, Integral University, IIRC-5, Kursi Road, Lucknow 226026, Uttar Pradesh, India; hayatbt@iul.ac.in
[4] Department of Biology, College of Sciences, University of Ha'il, P.O. Box 2440, Ha'il 81411, Saudi Arabia; naib.alshammari@uoh.edu.sa (N.A.); mo.saeed@uoh.edu.sa (M.S.)
[5] Department of Biology, College of Science, Princess Nourah Bint Abdulrahman University, P.O. Box 84428, Riyadh 11671, Saudi Arabia
[6] Department of Life Sciences and Biotechnology, C.S.J.M.U (Formerly Kanpur University), Kanpur -208024, Uttar Pradesh, India

*** Correspondencing authors**:
L.A.A.-K.; laalkeridis@pnu.edu.sa
R.S.;roleesh@gmail.com or roleesharma@csjmu.ac.in

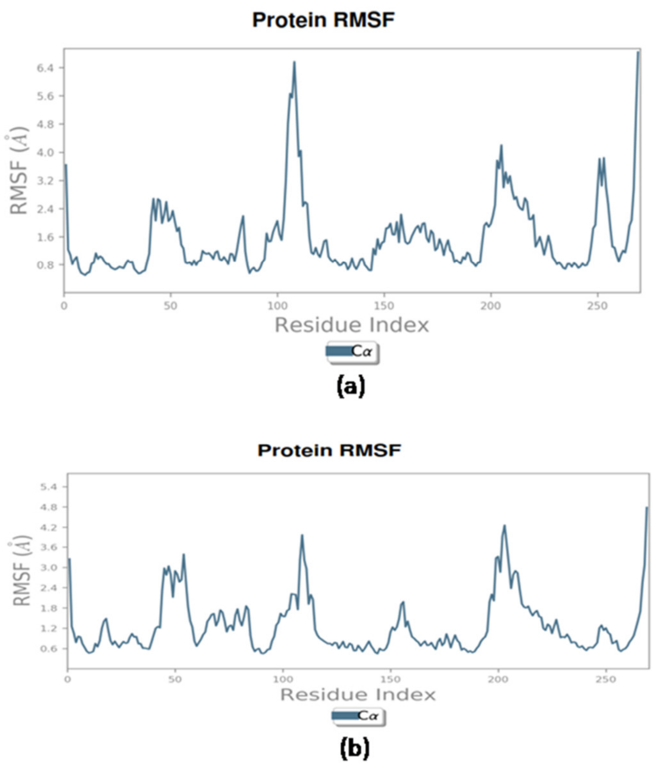

Figure S1. RMSF plot for the InhA with (a) Gravacridonediol (b) Triclosan

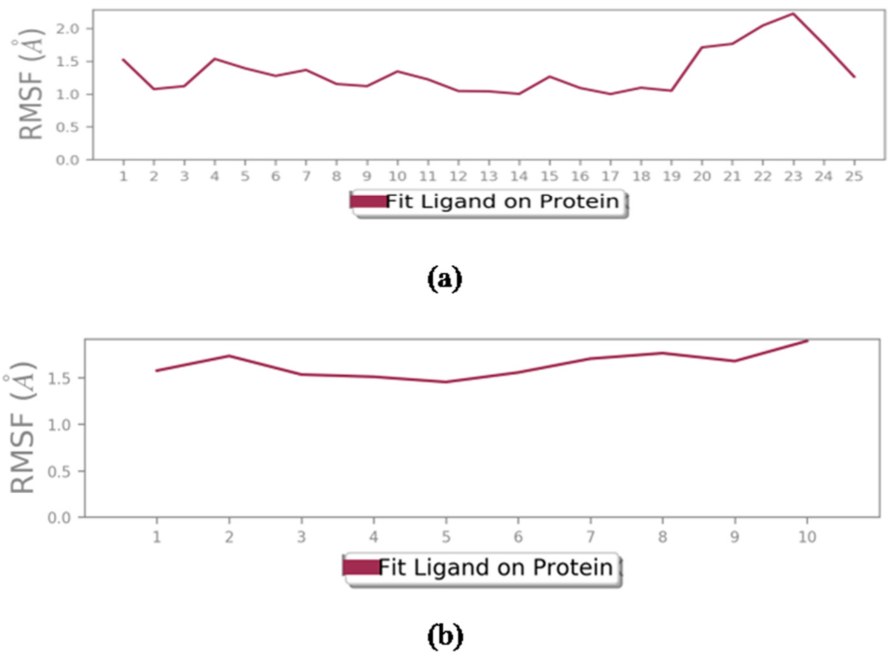

Figure S2. RMSF plot for (a) Gravacridonediol (b) Triclosan

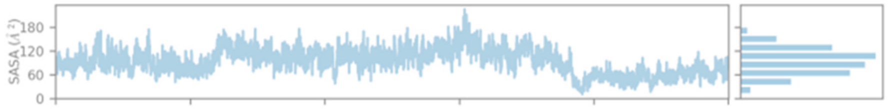

(a)

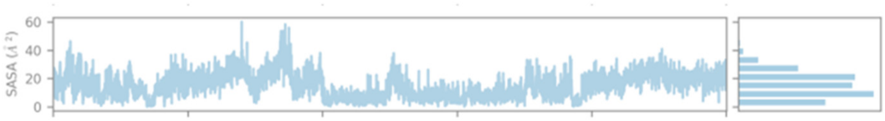

(b)

Figure S3. Solvent accessible surface area (SASA) of (a) Gravacridonediol (b) Triclosan

Table S1. Compilation of compounds.

S.no.	PUBCHEM ID	COMPOUND	CLASS
1.	26049	3-Carene	Terpenoids
2.	232703	α-Limonene diepoxide	Terpenoids
3.	638011	Citral	Terpenoids
4.	22311	Limonene	Terpenoid
5.	521268	Isolimonene	Terpenoids
6.	448438	Violaxanthin	Terpenoids
7.	52808998	Zeaxanthin	Terpenoids
8.	91354	Aromadendrene	Terpenoids
9.	363127	Taiwanin-c	Terpenoids
10.	22506525	Undecenol	Alcohol
11.	8194	Dodecanal	Alcohol
12.	15448	Methyl-n-nonyl-carbinol	Alcohol
13.	1268142	Nootkatone	Ketone
14.	17691	3-Methyl-2-cyclopenten-1-one	Ketones
15.	8163	Methyl-n-nonyl-ketone	ketone
16.	13187	Methyl-heptyl-ketone	Ketone
17.	449290	Trans-limonene oxide	Lipids (isoprenoids)
18.	87839	cis-Verbenol	Prenol lipid(onoterpenoids)
19.	5281235	β-Cryptoxanthin	Carotenoids
20.	442428	Naringin	Flavonoid
21.	442439	Neohesperidin	Flavonoid
22.	5282150	Rhoifolin	Flavonoid
23.	114627	Neoeriocitrin	Flavonoid
24.	16760075	Didymin	Flavanoid
25.	83489	Eriocitrin	Flavanoid
26.	10621	Hesperedin	Flavanoid
27.	442431	Narirutin	Flavanoid
28.	442456	Ponicirin	Flavanoid
29.	9851181	Isorhoifolin	Flavanoid
30.	5281613	Diosmin	Flavanoid
31.	69964214	Neodiosmin	Flavanoid
32.	72344	Nobiletin	Flavanoid
33.	145659	Sinenseti n	Flavanoid
34.	68077	Tangeretin	Flavanoid
35.	439533	Taxifolin	Flavanoid
36.	5280442	Acacetin	Flavanoid
37.	5281612	diosmetin	Flavonoids
38.	72276	(-) epicatechol	Flavonoide
39.	5280443	Apigenin	Flavonoide
40.	2775	Citropten	Coumarins
41.	10212	Imperatorin	Coumarins
42.	68279	Isopimpinellin	Coumarins
43.	98608	Phellopeterin	Coumarins
44.	5280460	Scopoletin	Coumarins

45.	8417	Scoparon	Coumarins
46.	65188	Xanthyletin	Coumarins
47.	128834	Chalepensin	Coumarins
48.	14133589	Gravelliferone	Coumarins
49.	10748	Herniarin	Coumarins
50.	68079	Isopimpinellin	Coumarins
51.	185756	Isorutarin	Coumarins
52.	6199	Psoralen	Coumarin
53.	182049	Rutacultin	Coumarins
54.	5281426	Umbelliferone	Coumarins
55.	4114	Xanthotoxin	Coumarins
56.	44146779	Rutaretin	Coumarins
57.	442149	Rutarin	Coumarins
58.	5321539	Suberenone	Coumarins
59.	107936	Gamma-fagarine	Furaquinolone
60.	135564263	Anisic-acid	Benzenoids
61.	460	Guaiacol	Phenol
62.	341	m-digallic acid	Phenol
63.	528594	protocatechuic	Phenol
64.	44144315	Pangelin	Phenol
65.	10227	Kokusaginine	Alkaloid
66.	5281842	Furacridone	Alkaloids
67.	11044132	Graveolinine	Alkaloid
68.	5317836	Gravacridonediol	Alkaloid
69.	21586648	Gravacridonetriol	Alkaloid
70.	353825	Graveoline	Alkaloid
71.	14133589	Gravelliferone-methyl-ether	Alkaloid
72.	336322	Ribalinidine	Alkaloid
73.	442931	Ribalinium	Alkaloid
74.	5281849	Rutacridone	Alkaloid
75.	5280805	Rutin	Alkaloid
76.	26948	Rutamarin	Alkaloid
77.	6590	Isobutyric-acid	Carboxylic acid
78.	1032	Propionic-acid	Carboxylic acid
79.	59129375	Methyl-butyric-acid	Lipids
80.	21160126	Pregeijerene	Hydrrocarbon

Table S2. Screening of compounds for drug-likeliness property.

S.no.	Name of compound	Pubchem Id	Molecular weight (g/mol) (≤500)	Xlog P3 (≤5)	H-bond donar (≤5)	H –bond acceptor (≤10)	Rotational bond (≤10)	n VIOLATION
1.	3-Carene	26049	136.23	2.8	0	0	0	0
2.	3-Methyl-2-cyclopenten-1-one	17691	96.13	0.5	0	1	0	0
3.	Trans-limonene oxide	449290	152.23	2.5	0	1	1	0

4.	α-Limonene diepoxide	232703	68.23	1.1	0	2	1	0
5.	(2E)-Undecenol	22506525	170.29	5	1	1	8	0
6.	Dodecanal	8194	184.323	4.9	0	1	10	0
7.	Nootkatone	1268142	218.33	3.9	0	1	1	0
8.	Isolimonene	521268	136.23	4	0	0	1	0
9.	Citral	638011	152.23	3	0	1	4	0
10.	Limonene	22311	136.23	3.4	0	0	1	0
11.	cis-Verbenol	87839	152.23	1.6	1	1	0	0
12.	Neoeriocitrin	114627	596.5	-0.9	9	15	6	3
13.	Naringin	442428	580.5	-0.5	8	14	6	3
14.	Neohesperidin	442439	610.6	-0.5	8	15	7	3
15.	Rhoifolin	5282150	578.5	-0.2	8	14	6	3
16.	β-Cryptoxanthin	5281235	552.9	12.3	1	1	10	2
17.	Violaxanthin	448438	600.9	9.8	2	4	10	2
18.	Zeaxanthin	52808998	568.9	10.9	2	2	10	2
19.	Aromadendrene	91354	204.35	4.7	0	0	0	0
20.	Didymin	16760075	594.6	-0.7	7	14	7	3
21.	Eriocitrin	83489	596.5	-1.4	9	15	6	3
22.	Hesperedin	10621	610.6	-1.1	8	15	7	3
23.	Narirutin	442431	580.5	-1.1	8	14	6	3
24.	Ponicirin	442456	594.6	-0.2	7	14	7	3
25.	Isorhoifolin	9851181	578.5	-0.8	8	14	6	3
26.	Diosmin	5281613	608.5	-0.8	8	15	7	3
27.	Neodiosmin	69964214	608.5	-0.3	8	15	7	3
28.	Nobiletin	72344	402.4	3	0	8	7	1
29.	Sinenseti n	145659	372.4	3	0	7	6	1
30.	Tangeretin	68077	372.4	3	0	7	6	1
31.	Taxifolin	439533	304.25	1.5	5	7	1	1
32.	Acacetin	5280442	284.26	2.1	2	5	2	0
33.	Scoparon	8417	206.19	1.9	0	4	2	0
34.	Xanthyletin	65188	228.34	2.8	0	3	0	0
35.	Citropten	2775	206.19	1.9	0	4	2	0
36.	Imperatorin	10212	270.28	3.4	0	4	3	0
37.	Isopimpinellin	68279	246.21	1.9	0	5	2	0
38.	Phellopeterin	98608	300.3	3.8	0	5	4	0
39.	Scopoletin	5280460	192.17	1.5	1	4	1	0
40.	Diosmetin	5281612	300.26	1.7	3	6	2	0
41.	m-digallic acid	341	322.22	1.1	6	9	4	0
42.	Protocatechuic	528594	496.9	-	0	4	10	1
43.	(-) Epicatechol	72276	290.27	0.4	5	6	1	0
44.	Apigenin	5280443	270.24	1.7	3	5	1	0
45.	Anisic-acid	135564263	317.8		1	5	6	1
46.	Chalepensin	128834	254.28	4.3	0	3	2	0
47.	Furacridone	5281842	265.26	3.7	1	4	0	0

48.	Gamma-fagarine	107936	229.23	2.9	0	4	2	0
49.	Gravacridonediol	5317836	341.4	2.3	3	6	2	0
50.	Gravacridonetriol	21586648	357.4	1.2	4	7	3	0
51.	Gravelliferone	14133589	298.4	5.4	1	3	4	0
52.	Gravelliferone-methyl-ether	14133589	298.4	5.4	1	3	4	0
53.	Graveoline	353825	279.29	3.1	0	4	1	0
54.	Graveolinine	11044132	279.29	3.9	0	4	2	0
55.	Guaiacol	460	124.14	1.3	1	2	1	0
56.	Herniarin	10748	176.17	1.9	0	3	1	0
57.	Isobutyric-acid	6590	88.11	0.8	1	2	1	0
58.	Isopimpinellin	68079	246.21	1.9	0	5	2	0
59.	Isorutarin	185756	424.4	0	5	10	4	0
60.	Kokusaginine	10227	259.26	2.6	0	5	3	0
61.	Methyl-heptyl-ketone	13187	142.24	3.1	0	1	6	0
62.	Methyl-butyric-acid	59129375	334.4	2.4	1	5	7	0
63.	Methyl-n-nonyl-carbinol	15448	172.31	4.5	1	1	8	0
64.	Methyl-n-nonyl-ketone	8163	170.29	4.1	0	1	8	0
65.	Pangelin	44144315	286.28	2.9	1	5	4	0
66.	Pregeijerene	21160126	162.27	3.4	0	0	0	0
67.	Propionic-acid	1032	74.08	0.3	1	2	1	0
68.	Psoralen	6199	186.16	2.3	0	3	0	0
69.	Ribalinidine	336322	275.3	1.6	2	5	0	0
70.	Ribalinium	442931	290.33	2.3	2	4	2	0
71.	Rutacridone	5281849	307.3	4.6	1	4	1	0
72.	Rutacultin	182049	274.31	3.8	0	4	4	0
73.	Rutamarin	26948	356.4	4.4	0	5	5	0
74.	Rutaretin	44146779	262.26	1.6	2	5	1	0
75.	Rutarin	442149	424.4	-0.2	5	10	4	0
76.	Rutin	5280805	610.5	-1.3	10	16	6	1
77.	Suberenone	5321539	244.24	2	0	4	3	0
78.	Taiwanin-c	363127	348.3	3.8	0	6	1	1
79.	Umbelliferone	5281426	162.14	1.6	1	3	0	0
80.	Xanthotoxin	4114	216.19	1.9	0	4	1	0

Table S3. ADMET profiling of all the screened compounds.

S.no.	Compound name	Toxicity		Adsorption				Distribution		Metabolism
		Mutagenicity (Ames test)	Carcinogenecity	HIA %	PCaco-2 (nm/sec)	PMDCK (nm/sec)	Pskin (nm/sec)	PPB %	BBB (Cbrain/Cblood)	CYP2D6
1.	3-Carene	Mutagenic	Non-carcinogenic	100	23.63	304.8	-1.46	100	5.5	Non-inhibitor
2.	3-Methyl-2-cyclopenten-1-one	Mutagenic	Non-carcinogenic	100	28.06	72.78	-2.57	88.46	0.7	Non-inhibitor
3.	Trans-limonene oxide	Mutagenic	Non-carcinogenic	100	53.63	183.7	-1.8	46.62	1.2	Non-inhibitor
4.	α-Limonene diepoxide	Mutagenic	Non-carcinogenic	100	57.6	28.79	-2.5	54.15	0.23	Non-inhibitor
5.	(2E)-Undecenol	Non-mutagenic	Carcinogenic	100	35.43	123.7	-0.7	100	9.3	Non-inhibitor
6.	Dodecanal	Non-mutagenic	Carcinogenic	100	45.7	108.504	-0.7	100	12.08	Non-inhibitor
7.	Nootkatone	Mutagenic	Carcinogenic	100	15.9	219.01	-0.89	100	2.8	Non-inhibitor
8.	Isolimonene	Mutagenic	Non-carcinogenic	100	23.49	261.53	-0.8	100	7.4	Non-inhibitor
9.	Citral	Mutagenic	Non-carcinogenic	100	13.96	252.255	-0.9	100	2	Non-inhibitor
10.	Limonene	Mutagenic	Non-carcinogenic	100	23.63	238.43	-0.8	100	8.2	Non-inhibitor
11.	cis-Verbenol	Mutagenic	Carcinogenic	100	25.95	103.25	-2.3	100	3.4	Non-inhibitor
12.	Aromadendrene	Mutagenic	Non-carcinogenic	100	+---	41.9	-0.9	100	11.36	Non-inhibitor
13.	Nobiletin	Mutagenic	Non-carcinogenic	99.07	54.02	0.06	-3.6	84.85	0.02	Non-inhibitor
14.	Sinenseti n	Mutagenic	Non-carcinogenic	98.8	51.22	0.06	-3.5	86.24	0.02	Non-inhibitor
15.	Tangeretin	Mutagenic	Non-carcinogenic	98.8	53.6	0.62	-3.4	87.17	0.02	Non-inhibitor
16.	Taxifolin	Mutagenic	Non-carcinogenic	60.16	3.4	9.56	-4.4	95.16	0.1	Non-inhibitor
17.	Acacetin	Mutagenic	Non-carcinogenic	93.04	12.79	20.23	-3.3	90	0.15	Non-inhibitor
18.	Scoparon	Mutagenic	Non-carcinogenic	97.8	2.7	119.04	-3	55.99	1.8	Non-inhibitor
19.	Xanthyletin	Mutagenic	Non-carcinogenic	98.9	24.63	78.07	-2.5	86.04	1.39	Non-inhibitor
20.	Citropten	Mutagenic	Non-carcinogenic	97.85	46.92	52.61	-3	78.49	1.81	Non-inhibitor
21.	Imperatorin	Mutagenic	Carcinogenic	97.7	56.7	228.7	-3.1	94.7	2.7	Non-inhibitor
22.	Isopimpinellin	Mutagenic	Non-carcinogenic	98.31	47.6	170.7	-3.8	88.9	1.6	Non-inhibitor
23.	Phellopterin	Mutagenic	Carcinogenic	98.13	55.88	185.43	-3.3	92.9	0.3	Non-inhibitor
24.	Diosmetin	Mutagenic	Non-carcinogenic	88.18	7.02	23.85	-4.1	90.16	0.2	Non-inhibitor

25.	Protocatechuic	Non-mutagenic	Carcinogenic	98.3	22.7	0	-3	100	15.8	Non-inhibitor
26.	(-) Epicatechol	Mutagenic	Carcinogenic	66.7	0.6	44.3	-4.2	100	0.3	Non-inhibitor
27.	Apigenin	Mutagenic	Non-carcinogenic	88.1	10.5	44.3	-4.1	97.2	0.5	Non-inhibitor
28.	Chalepensin	Mutagenic	Carcinogenic	97.99	50.58	60.97	-2.44	95.7	2.06	Non-inhibitor
29.	Furacridone	Mutagenic	Non-carcinogenic	95.7	9.91	35.22	-3.66	89.55	1.11	Non-inhibitor
30.	Gamma-fagarine	Mutagenic	Non-carcinogenic	97.71	55.93	61.55	-3.74	82.37	2.44	Non-inhibitor
31.	Gravacridonediol	Non-mutagenic	Non-carcinogenic	100	19.17	45.96	-4.02	79.22	0.26	Non-inhibitor
32.	Graveoline	Non-mutagenic	Non-carcinogenic	83.77	19.23	5.39	-4.52	67.98	0.12	Non-inhibitor
33.	Graveolinine	Mutagenic	Non-carcinogenic	97.83	56.13	37.77	-3.47	89.96	0.04	Non-inhibitor
34.	Guaiacol	Mutagenic	Non-carcinogenic	96.47	29.44	362.86	-1.9	99.18	0.9	Non-inhibitor
35.	Helioxanthin	Mutagenic	Carcinogenic	97.75	29.6	112.05	-4.23	89.81	2.75	Non-inhibitor
36.	Herniarin	Mutagenic	Non-carcinogenic	99.14	40.68	59.04	-2.35	69.85	1.82	Non-inhibitor
37.	Isobutyric-acid	Mutagenic	Non-carcinogenic	86.77	20.71	127.13	-2.64	75.2	1.13	Non-inhibitor
38.	Isorutarin	Non-mutagenic	Carcinogenic	44.22	12.16	0.44	-4.7	49.12	0.04	Non-inhibitor
39.	Isopimpinellin	Mutagenic	Non-carcinogenic	98.32	47.63	170.76	-3.81	88.9	1.67	Non-inhibitor
40.	Isovaleric-acid	Mutagenic	Non-carcinogenic	89.09	20.83	53.42	-2.4	82.25	1.19	Non-inhibitor
41.	Kokusaginine	Mutagenic	Non-carcinogenic	97.86	56.29	64.18	-3.95	79.93	1.66	Non-inhibitor
42.	Methyl-butyric-acid	Mutagenic	Carcinogenic	94.46	16.86	77.1	-2.74	95.73	0.22	Non-inhibitor
43.	Methyl-heptyl-ketone	Mutagenic	Non-carcinogenic	100	26.76	238.78	-1.41	100	1.34	Non-inhibitor
44.	Methyl-n-nonyl-ketone	Non-mutagenic	Carcinogenic	100	44.9	204.49	-1	100	6.55	Non-inhibitor
45.	Pangelin	Mutagenic	Non-carcinogenic	96.37	11.68	59.15	-3.3	86.31	0.24	Non-inhibitor
46.	Pregeijerene	Mutagenic	Non-carcinogenic	100	23.63	42.35	-0.7	100	10.95	Non-inhibitor
47.	Propionic-acid	Mutagenic	Non-carcinogenic	83.59	19.18	114.9	-2.85	63.19	0.93	Non-inhibitor
48.	Psoralen	Mutagenic	Non-carcinogenic	98.43	37.75	67.47	-3.52	91.07	2.37	Non-inhibitor
49.	Quercetin	Mutagenic	Non-carcinogenic	63.49	3.41	13.35	-4.43	93.24	0.17	Non-inhibitor
50.	Ribalinidine	Non-mutagenic	Carcinogenic	92.93	22.48	39.14	-3.63	64.79	0.68	Non-inhibitor
51.	Ribalinium	Non-mutagenic	Carcinogenic	93.07	14.54	10.19	-3.41	27.86	0.47	Substrate
52.	Rutacridone	Mutagenic	Non-carcinogenic	95.74	35.63	8.75	-3.28	89.6	0.87	Non-inhibitor
53.	Rutacultin	Mutagenic	Non-carcinogenic	98.04	48.67	212.52	-2.41	88.53	0.12	Non-inhibitor

54.	Rutamarin	Mutagenic	Carcinogenic	98.37	44.51	0.07	-1.87	90.31	0.03	Non-inhibitor
55.	Rutaretin	Non-mutagenic	Non-carcinogenic	90.96	5.84	253.44	-3.18	72.17	0.59	Non-inhibitor
56.	Rutarin	Non-mutagenic	Carcinogenic	44.39	14.36	0.3	-4.44	42.29	0.04	Non-inhibitor
57.	Suberenone	Mutagenic	Non-carcinogenic	98.19	31.65	52.56	-3.22	82.73	0.33	Non-inhibitor
58.	Umbelliferone	Mutagenic	Non-carcinogenic	94.1	19.5	55.9	-2.5	42.39	0.5	Non-inhibitor
59.	Xanthotoxin	Mutagenic	Non-carcinogenic	98.1	43.37	360.7	-3.6	93.04	2	Non-inhibitor
